# Comparative genomic insights into the genus *Pantoea*: genetic determinants of ecological lifestyle diversity and plant growth–promoting potential

**DOI:** 10.1007/s11274-025-04780-2

**Published:** 2026-01-17

**Authors:** Felipe F. Rimes-Casais, Francisnei Pedrosa-Silva, Thiago Motta Venancio

**Affiliations:** Laboratório de Química e Função de Proteínas e Peptídeos (LQFPP), Centro de Biociências e Biotecnologia, Universidade Estadual do Norte Fluminense Darcy Ribeiro, Campos dos Goytacazes, Brazil

**Keywords:** Comparative genomics, Pantoea, PGPB, Lifestyle, Pan-GWAS

## Abstract

*Pantoea* is a genus of Gram-negative bacteria isolated from diverse environments. Over time, it has drawn considerable attention for its potential to promote plant growth. However, its biotechnological application is complicated by high genomic plasticity, which underlies both its beneficial traits and its ability to cause disease in a wide range of plants, as well as occasional opportunistic infections in humans, raising biosafety concerns. In this study, we conducted a comparative genomic analysis of all publicly available *Pantoea* genomes. Our goals were to refine taxonomic classifications and to identify genes linked to biotechnological potential, virulence, and antibiotic resistance, thereby clarifying lifestyle strategies within the genus. We found that plant growth-promoting genes are widely conserved, particularly those involved in phosphate solubilization, phytohormone biosynthesis, and siderophore production. In contrast, traits such as nitrogen fixation and ACC deaminase activity were restricted to specific species. The resistome analysis revealed intrinsic resistance mechanisms conserved across the genus, primarily involving diverse efflux pump families and β-lactamases conferring resistance to cephalosporins. In parallel, the pan-GWAS highlighted lifestyle-defining genetic markers, including the *hrp/hrc* genes encoding type III secretion system components, *pepM* (phosphoenolpyruvate mutase) associated with the production of a phytotoxin, and *ibeB*, an invasin linked to clinical infections. Together, our findings underscore both the biotechnological potential of *Pantoea* and the importance of genetic markers for distinguishing beneficial from pathogenic lifestyles, supporting the safe application of selected strains in biotechnology.

**Supplementary Information:**

The online version contains supplementary material available at 10.1007/s11274-025-04780-2.

## Introduction

*Pantoea* is a genus of Gram-negative, motile, rod-shaped bacteria belonging to the family Erwiniaceae. The genus name, meaning “of all types and sources”, reflects its broad ecological distribution and diversity. Members of *Pantoea* are frequently isolated from plants, where they are well-known colonizers with strong competitive ability and remarkable adaptability to diverse hosts (Crosby et al. 2023). Beyond plants, these bacteria have also been recovered from soil, water, in commensal interactions with insects, and in urban environments (Walterson and Stavrinides 2015), underscoring their versatile and wide-ranging ecological lifestyle.

Over the past decades, *Pantoea* has attracted increasing attention for its biotechnological potential, particularly in the context of plant growth promotion. Several strains act as plant growth-promoting bacteria (PGPB) through mechanisms such as phosphate solubilization, nitrogen fixation, phytohormone production, and siderophore synthesis (Quecine et al. 2012; Nascimento et al. 2020; Lv et al. 2022). In addition, they can enhance plant resilience under stress, including mitigating drought-induced water stress (Luziatelli et al. 2020). The genus biotechnological potential also extends to its application as a bioinoculant and biocontrol agent, antagonizing phytopathogens and stimulating plant innate immunity (Duchateau et al. 2024). These characteristics underscore the genus potential for sustainable crop management.

Despite this promise, the genus also harbors pathogenic species. *Pantoea stewartii*, for example, causes Stewart’s wilt in sweet corn (Ibrahim et al. 2022), while *Pantoea ananatis* is responsible for rice grain discoloration (Doni et al. 2021). Pathogenic strains of *Pantoea agglomerans* induce gall formation in *Gypsophila* and beet (Lorenzi et al. 2022), and additional cases of pathogenic isolates have been reported from other plants such as onion and eucalyptus (Vahling-Armstrong et al. 2016; Duan et al. 2025).

Beyond plants, *Pantoea* can occasionally infect humans, although such cases are much rarer and less studied. Reported clinical manifestations include nosocomial pneumonia, urinary tract infections, bacteremia, and wound infections (Ruan et al. 2022; Wang et al. 2024).

This duality—beneficial versus pathogenic lifestyles—poses a critical challenge for the safe application of *Pantoea* in biotechnology. Phenotypic plasticity complicates the screening of strains for safe use as bioinoculants, a challenge shared with other bacterial genera (Tariq et al. 2022). Moreover, despite advances in understanding the mechanisms of phytopathogenicity, the genetic basis underlying the diversity of lifestyles within the genus remains incompletely characterized.

In this study, we address this knowledge gap using comparative genomics of the *Pantoea* genus. We identify species with the greatest biotechnological potential, particularly as biofertilizers, and assess their safety by examining genetic determinants associated with phytopathogenic and clinical lifestyles.

## Methods

### Genome curation and reclassification

A total of 1,286 *Pantoea* spp. genomes available in the GenBank database (accessed February 10, 2025) were retrieved. Genome quality was assessed using CheckM v1.0.13 (Parks et al. 2015), applying a minimum completeness threshold of 90% and a maximum contamination of 10%. Genomes containing more than 500 contigs were discarded. To remove redundancy, in-house scripts based on Mash v2.3 (Ondov et al. 2016) were used to cluster genomes with a Mash distance below 0.05 (~ 99.95% ANI) (Passarelli-Araujo et al. 2022). Within each cluster, the genome with the highest N50 was retained as the representative. Subsequently, ANI comparisons were then performed using ANIm (MUMmer-based alignment) implemented in pyANI v0.2.7 (Pritchard et al. 2016).

### Gene prediction, mobile elements, and phylogeny

Protein-coding sequences were predicted using Prokka v1.13.4 (Seemann 2014). Mobile genetic elements were identified through plasmid prediction with PlasForest v1.4 (Pradier et al. 2021) and genomic island detection with IslandViewer 4 (http://www.pathogenomics.sfu.ca/islandviewer/, accessed March 27, 2025) (Bertelli et al. 2017). Core single-copy orthologs were identified using OrthoFinder v3.1.0 (Emms and Kelly 2019). These orthologs were aligned with MAFFT (Katoh and Standley 2013) and used to reconstruct a maximum likelihood phylogeny with IQ-TREE v2.4.0 (Nguyen et al. 2015). The best-fitting model (Q.plant + F + R10) was determined with ModelFinder, and clade support was assessed with 1,000 bootstrap replicates. Phylogenetic trees were visualized using iTOL v7.2.1 (Letunic and Bork 2019).

### Super-pangenome analysis and pan-GWAS

The super-pangenome of the genus *Pantoea* was reconstructed using Roary v3.13.0 (Page et al. 2015), applying an 80% identity threshold for ortholog clustering. A genome-wide association study (GWAS) was then conducted with Scoary v1.6.16 (Brynildsrud et al. 2016), using lifestyle categories (environmental, phytopathogenic, clinical) as phenotypes. Input data included the gene presence/absence matrix from Roary and the previously generated phylogenetic tree to account for population structure. To reduce phylogenetic bias, Scoary’s permutation test was applied with 1,000 randomizations of lifestyle labels. Statistical significance was determined by adjusting p-values with the Benjamini–Hochberg method. Genes were considered significant when meeting three criteria: specificity > 90%, adjusted *p* ≤ 0.05, and empirical *p* ≤ 0.05.

### Mining of genes of interest

Genes functionally related to host interaction or antibiotic resistance were identified using Usearch v11.0.667 (Zhou et al. 2024), with minimum thresholds of 50% identity and 80% coverage. Searches were performed sequentially against: (1) an in-house curated database of plant growth-promoting genes; (2) the Virulence Factors of Pathogenic Bacteria Database (VFDB) (Liu et al. 2022); (3) the Pathogen Host Interaction database (PHI-base) (Urban et al. 2025); and (4) the Comprehensive Antibiotic Resistance Database (CARD) (Alcock et al. 2023). The latter three databases were used to identify virulence- and resistance-associated genes, representing the virulome and resistome of the genus. All databases were accessed in May 2025.

## Results

### Genome filtering, species delimitation, and phylogeny

To enable robust comparative analyses and genetic mining, we first curated the genomic dataset, ensuring quality, removing redundancy, and clarifying taxonomic assignments. In total, 1,286 genomes of *Pantoea* spp. were retrieved from the NCBI GenBank database (February 2025). From this initial set, we selected genomes containing fewer than 500 contigs. We then assessed assembly completeness by calculating the percentage of expected single-copy genes, retaining only those with > 90% completeness. In addition, we screened for multiple copies of single-copy genes and excluded genomes with > 10% contamination. After applying these quality filters, 1,151 genomes were retained.

Redundancy was reduced using Mash, which clustered genomes with pairwise distances below 0.05 (corresponding to 99.95% ANI), identifying 506 duplicate assemblies. Within each cluster, the assembly with the highest contiguity (N50) was selected as the representative genome. This process resulted in a final dataset of 645 high-quality, non-redundant genomes that were used for subsequent analyses, Table S1 lists all downloaded genomes, indicating duplicates and reasons for exclusion.

Species delimitation was performed using an all-vs-all ANI analysis. Genomes sharing ≥ 95% ANI (Bobay 2020), the accepted threshold for bacterial species boundaries, were grouped together. This analysis identified 189 necessary reclassifications, the majority (80.4%) corresponding to previously unclassified strains. Interestingly, some discrepancies involved type strains themselves—for example, the type strain of *P. leporis* shares 98.5% ANI with *P. endophytica*, which also possesses its own type strain. Additional cases of misassignment are provided in Table S2.

**Fig. 1:**
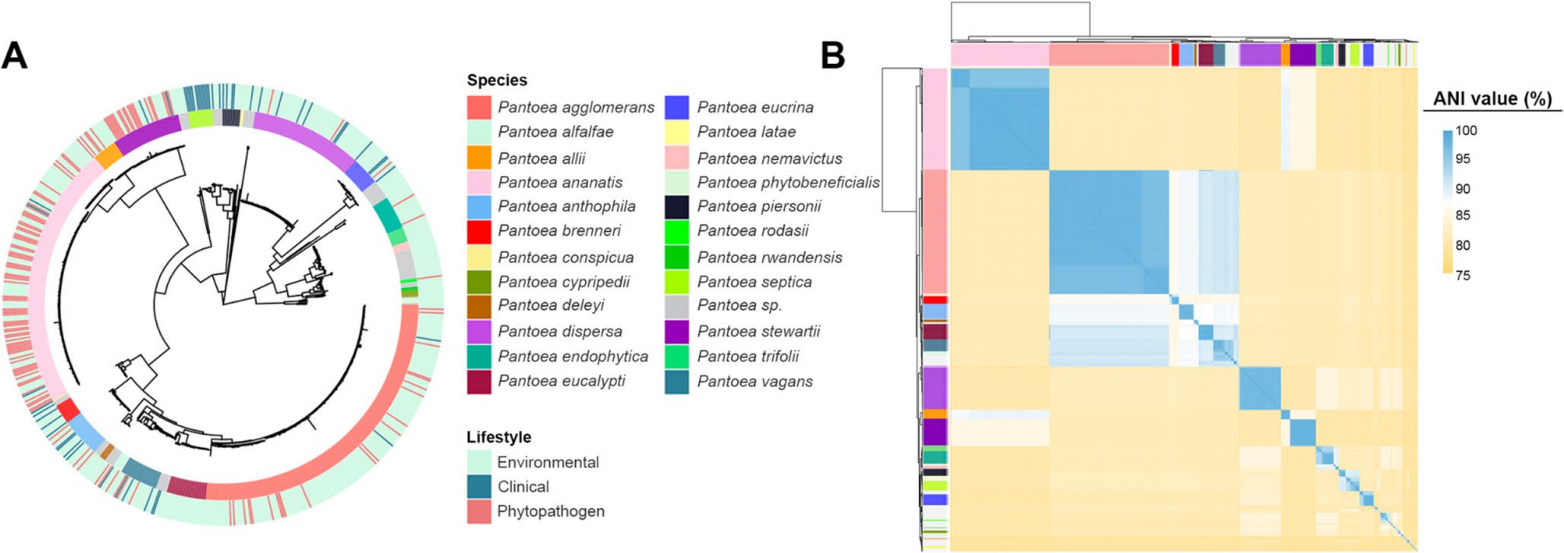
Phylogeny and diversity of the *Pantoea* genus. (**A**) Maximum likelihood phylogenetic tree of *Pantoea* genomes, indicating species and their associated lifestyles. Reconstruction was performed with IQ-TREE. (**B**) ANI-based clustering showing 23 genomic groups defined by > 95% identity

A maximum likelihood phylogeny was then reconstructed using single-copy orthologs genes identified by OrthoFinder (see Methods for details). The reconstruction resolved 23 major groups, each containing a type strain. These phylogenetic clusters were congruent with the ANI-based species boundaries, reinforcing the robustness of both approaches for taxonomic delimitation within the genus *Pantoea* (Fig. 1).

Species distribution across lifestyles revealed distinct trends. The most frequently represented phytopathogenic taxa were *P. ananatis*, *P. stewartii*, *P. allii*, and *P. agglomerans*, consistent with their recurrent association with crop diseases. In contrast, strains isolated from clinical settings were predominantly assigned to *P. septica*, *P. piersonii*, *P. brenneri*, and *P. anthophila*. The remaining species were mostly of environmental origin, commonly associated with plants, and lacked records of pathogenicity or deleterious interactions (Fig. 1).

### Plant growth-promoting potential of ***Pantoea***

After defining genomic groups and reclassifying the genomes, we examined the repertoire of genes with potential biotechnological relevance, focusing on those associated with plant growth promotion. Strains of *Pantoea* have been increasingly recognized as candidates in agricultural biotechnology, given their documented effects on plant development (Walterson and Stavrinides 2015). To explore this potential, we screened the genus for classical PGPB traits, including genes involved in nitrogen fixation, phosphate solubilization, phytohormone production, siderophore synthesis, and ethylene modulation. The genes used for genome mining and their predicted functions are listed in Table S3.

**Fig. 2:**
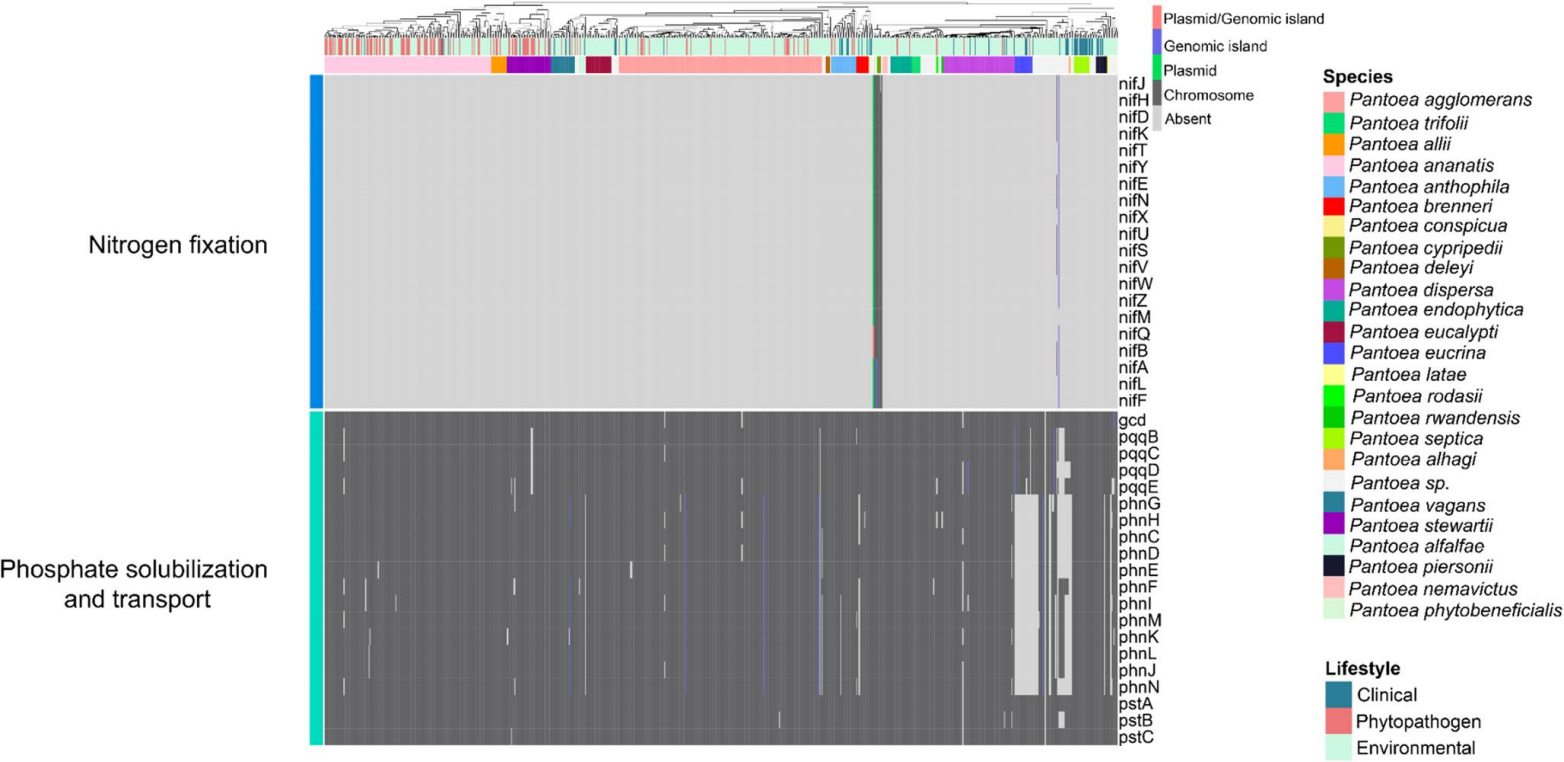
Presence–absence heatmap of genes associated with nitrogen fixation, phosphate solubilization and transport. Columns correspond to individual genomes and are annotated with species names and their respective lifestyles. The occurrence of the same gene in different genomic regions is represented by distinct colors

**Fig. 3:**
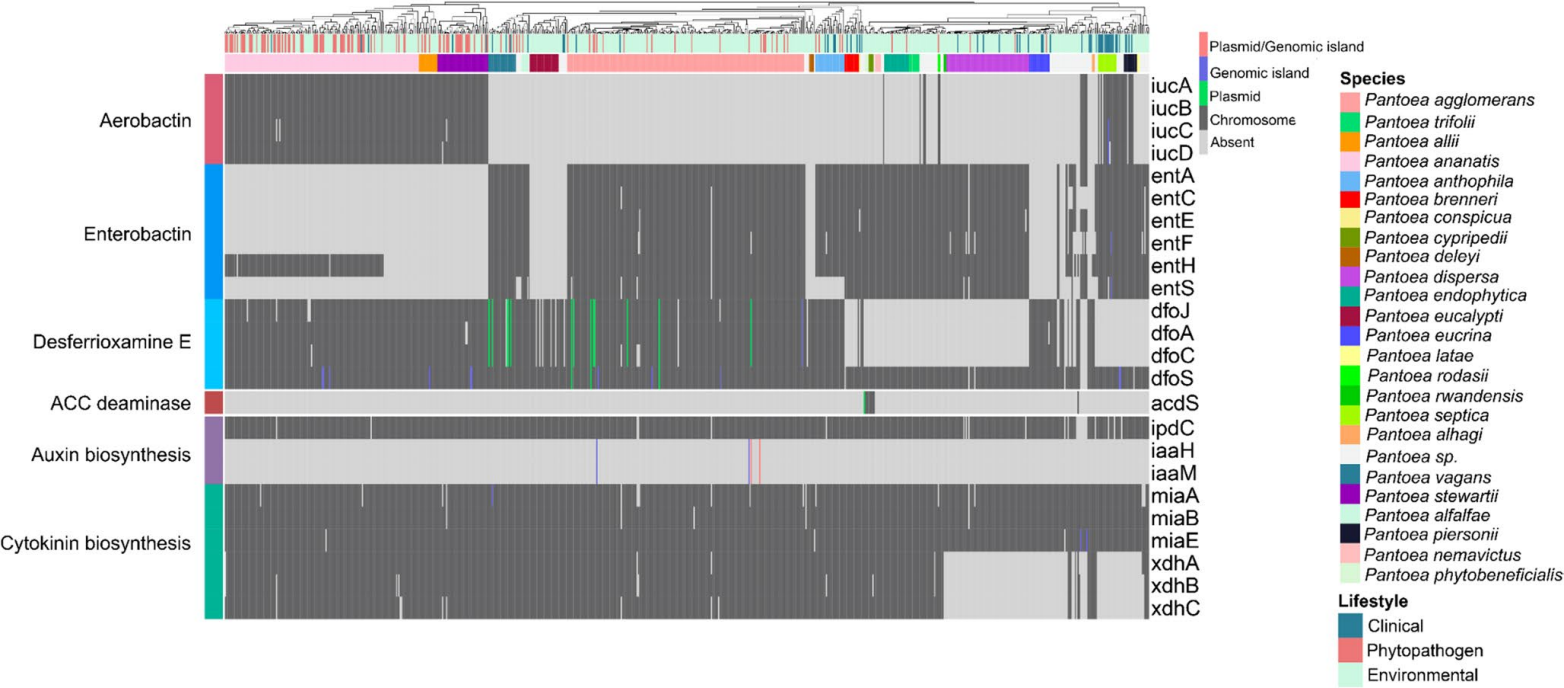
Presence–absence heatmap of genes associated with siderophore biosynthesis, phytohormone production, and ethylene level modulation. Columns represent individual genomes and are annotated with species and their respective lifestyles. Distinct colors indicate the occurrence of the same gene in different genomic regions

Figures 2 and 3 show presence–absence profiles of PGPB-related genes, revealing a broadly homogeneous distribution across *Pantoea* lifestyles. Notably, even phytopathogenic and clinical strains harbor genes traditionally linked to plant growth promotion. These findings indicate that the mere presence of such genes is insufficient to distinguish beneficial from pathogenic lifestyles within the genus. A more detailed discussion of the principal PGPB-related genes identified is provided below.

### Nitrogen fixation and phosphate solubilization

A classical trait of PGPB is the ability to fix atmospheric nitrogen, converting it into forms readily assimilable by plants (Sepp et al. 2023). This function is mediated by the *nif* operon, which encodes approximately 20 proteins involved in nitrogen uptake, biosynthesis of the iron–molybdenum cofactor, reduction of nitrogen to ammonia, and regulatory functions.

All *nif* genes were detected exclusively in *P. cypripedii*, *P. phytobeneficialis*, and two unclassified strains, forming a monophyletic group (Fig. 2). In one of these strains, the *nif* cluster is located on a plasmid that also harbors the genes *acdS* and *acdR* (associated with ACC deaminase activity) (Fig. 3), as well as genes related to iron transport, jasmonic acid biosynthesis, and glucose-1-dehydrogenase. In *P. cypripedii* and *P. phytobeneficialis*, the *nif* genes are chromosomally encoded and syntenic, suggesting chromosomal integration after plasmid acquisition through horizontal gene transfer (HGT). Thus, nitrogen fixation in *Pantoea* is circumscript to a small monophyletic group that likely acquired such genes through a common HGT event.

Phosphate solubilization represents another crucial PGPB trait, given the limited availability of plant-assimilable phosphorus in most soils (Olanrewaju et al. 2017). Genes related to inorganic phosphate solubilization, such as *gcd* (glucose-1-dehydrogenase) and its cofactor pqq (pyrroloquinoline quinone), which mediate gluconic acid (GA) synthesis, were identified. GA promotes soil acidification, thereby releasing phosphate ions for plant uptake. Genes associated with GA production were consistently detected in chromosomes of all *Pantoea* species, suggesting that phosphate solubilization is an intrinsic feature of the genus.

In addition, the *phn* operon, which encodes phosphonatase—an enzyme responsible for the degradation of organophosphonate compounds that can also serve as phosphorus sources—was detected in most species. Notable exceptions included *P. eucrina*, *P. coffeiphila*, and several unclassified strains.

### Stress control, phytohormone production, and siderophore biosynthesis

Under biotic and abiotic stress, plants often accumulate ethylene, a key hormone that regulates various physiological processes but, at high levels, inhibits root growth and root hair formation (Ahemad and Kibret 2014). Certain endophytic bacteria mitigate these effects by producing 1-aminocyclopropane-1-carboxylate (ACC) deaminase, which metabolizes ACC, the direct precursor of ethylene in the Yang cycle. The gene encoding this enzyme, *acdS*, was identified in only two species, *P. cypripedii* and *P. phytobeneficialis* (Fig. 3).

Auxin, particularly indole-3-acetic acid (IAA), is another phytohormone central to plant development, especially in root growth and cell differentiation (Tang et al. 2023). Several bacterial pathways synthesize IAA, named according to their intermediates. In *Pantoea*, the predominant pathway is the indole-3-pyruvic acid (IPyA) route, catalyzed by indole-3-pyruvate decarboxylase, encoded by *ipdC*. This gene was detected in all species, located on chromosomes, indicating an ancestral and conserved origin. In contrast, the indole-3-acetamide (IAM) pathway, dependent on *iaaM* and *iaaH*, which encode tryptophan monooxygenase and indole-3-acetamide hydrolase, respectively, was found only in four *P. agglomerans* strains, including the pathogenic pathovars *P. agglomerans* pv. *betae* and pv. *gypsophila*, both associated with gall formation. In these strains, IAM genes occur within genomic islands and are often linked to virulence determinants such as the *hrp/hrc* operon, which encodes components of the type III secretion system (T3SS) and the induction of tumor formation (Barash and Manulis-Sasson 2009). These findings are consistent with previous reports showing that the IAM pathway is characteristic of phytopathogens (Ahemad and Kibret 2014), whereas the IPyA route has a broader distribution across environmental, pathogenic, and clinical strains, making its role less specific to lifestyle.

In addition to auxin, genes associated with the production of other phytohormones, such as cytokinin—a key regulator of cell division, seed germination, and seed development—were also identified (Orozco-Mosqueda et al. 2023). This pathway involves genes related to tRNA modification and recycling (*miaA*, *miaB*, and *miaE*), as well as subunits of xanthine dehydrogenase (*xdhA*, *xdhB*, and *xdhC*) (Rocha et al. 2023). Some species, including *P. dispersa*, *P. eucrina*, *P. latae*, *P. septica*, and *P. piersonii*, lacked the *xdhABC*. However, these species harbor *yagTSR*, which also encodes a xanthine dehydrogenase (Neumann et al. 2009), enabling cytokinin biosynthesis (Hossain 2024).

Siderophores, iron-chelating molecules produced by rhizospheric and endophytic bacteria, enhance iron availability to plants and contribute to pathogen suppression by outcompeting fungal siderophores (Olanrewaju et al. 2017). Three siderophore types were detected in *Pantoea*: enterobactin (*ent* operon), desferrioxamine E (DFO-E, *dfo*), and aerobactin (*iuc*). While enterobactin and DFO-E-related genes are broadly distributed with occasional losses and no clear lifestyle correlation, aerobactin genes show a more restricted pattern, occurring mainly in *P. ananatis*, *P. allii*, and *P. stewartii* (primarily phytopathogens), and *P. septica* (a clinical species). These results support previous observations that an enterobactin-like cluster was present in the last common ancestor of the genus, with subsequent losses in certain species and independent acquisitions of other siderophore clusters (e.g. aerobactin), via HGT (Soutar and Stavrinides 2018).

### Antibiotic resistance potential

For the safe implementation of *Pantoea* strains with high biotechnological potential, it is crucial to assess their resistome, i.e., the set of genes associated with antibiotic resistance, in order to avoid establishing genetic reservoirs that could facilitate the spread of clinically relevant resistance determinants (Larsson and Flach 2022). Our analysis revealed that the two predominant resistance mechanisms in the genus are efflux pump-mediated export and antibiotic inactivation by enzymes.

**Fig. 4:**
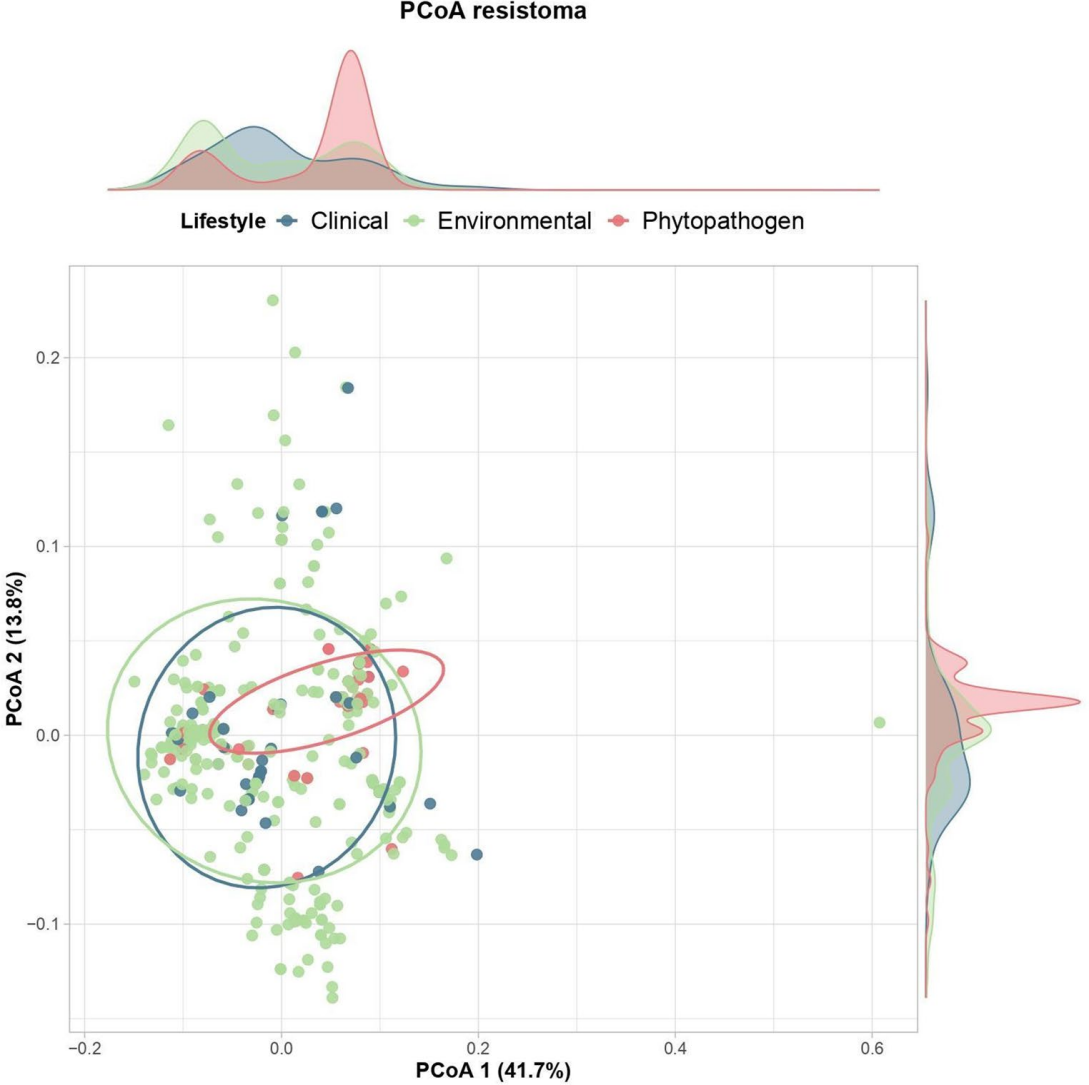
Principal Coordinate Analysis (PCoA) of dissimilarities in antibiotic resistance gene content across *Pantoea* genomes with different lifestyles. Density plots along the axes illustrate the distribution of genomes relative to each coordinate. Ellipses represent the 90% confidence interval for each lifestyle group, indicating the degree of dispersion and overlap among their resistome profiles

The distribution of resistance genes does not follow a lifestyle-specific pattern (Fig. 4), with clinical strains not showing a distinct repertoire that could discriminate them from environmental, endophytic, or other isolates. It is noteworthy that although some resistance genes were associated with mobile elements, no resistance islands carrying genes related to last-generation antibiotics, such as *bla*_CTX−M−15_ (third-generation cephalosporins) (Darby et al. 2023), *bla*_OXA−48−_like (carbapenems) (Hendrickx et al. 2021), and *qnr* (fluoroquinolones) (Amereh et al. 2023), were observed.

This suggests that the *Pantoea* resistome does not reflect recent adaptations driven by strong selective pressures in clinical environments, as seen in bacteria involved in hospital outbreaks (Sanikhani et al. 2024). Thus, the resistance repertoire found appears to represent intrinsic genetic characteristics of the genus rather than recent acquisitions of new mechanisms against modern antibiotics.

#### Efflux pumps and antibiotic inactivation

**Fig. 5:**
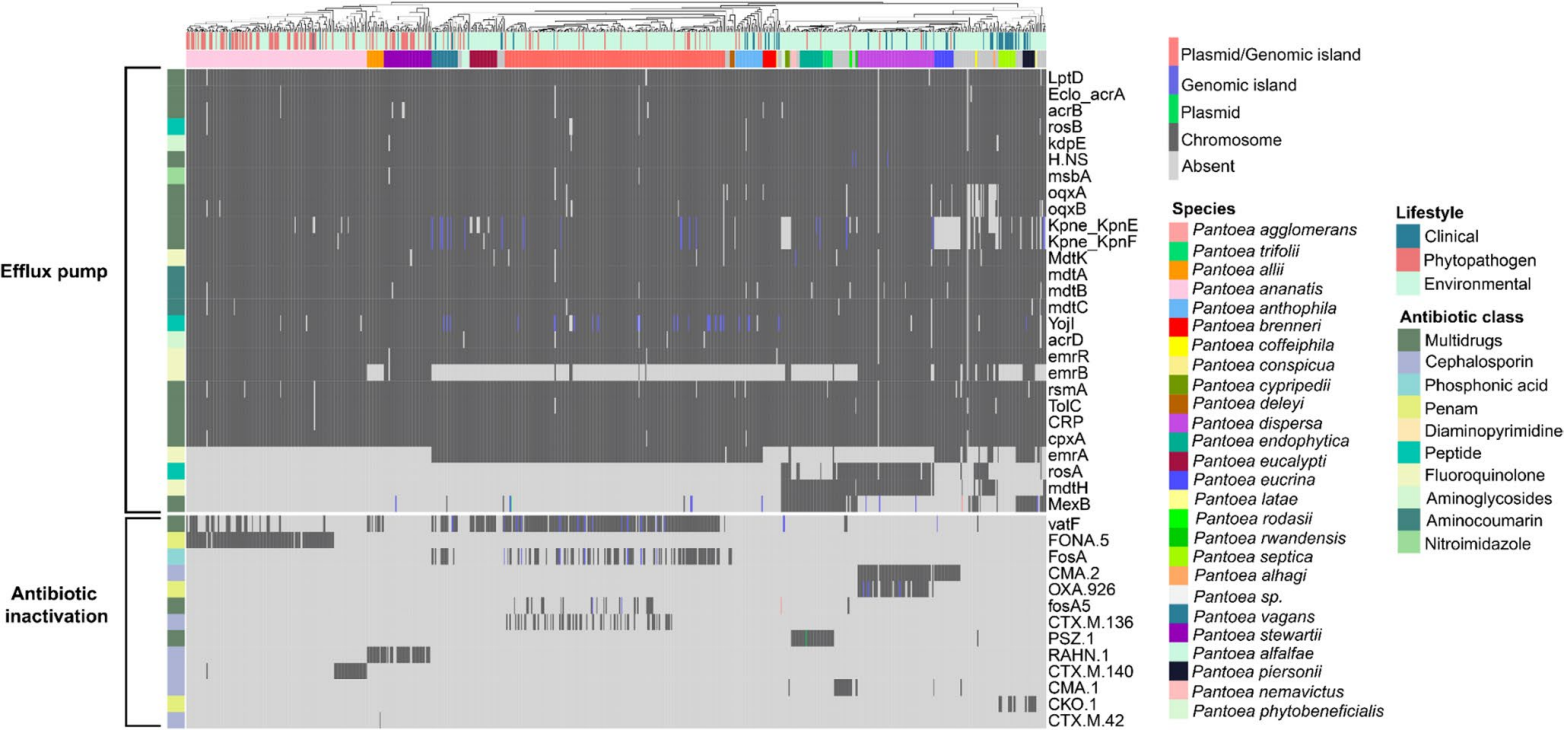
Presence–absence heatmap of genes related to efflux pumps and antibiotic inactivation. Genes are grouped according to the antibiotic classes to which they confer resistance, while genomes are labeled by species and lifestyle

Efflux pumps are key bacterial defense systems that expel toxic compounds, including antibiotics, thereby enhancing survival in adverse environments (Huang et al. 2022). A wide diversity of efflux pump genes is distributed across the analyzed strains, many of which are linked to the export of multiple antibiotic classes (Fig. 5). This pattern indicates that resistance mechanisms are intrinsic to the genus.

Among the efflux pump superfamilies identified, the most prominent were RND (Resistance-Nodulation-Division), MFS (Major Facilitator Superfamily), and SMR (Small Multidrug Resistance). The *acrAB* and *oqxAB* complexes, belonging to the RND superfamily, mediate the extrusion of several antibiotics, particularly cephalosporins and fluoroquinolones. These systems confer resistance to second- and third-generation fluoroquinolones as well as third- and fourth-generation cephalosporins in clinical pathogens such as *E. coli* and *K. pneumoniae* (Huguet et al. 2013; Bialek-Davenet et al. 2015). Additional relevant genes were also detected, including *rosAB* (MFS), associated with resistance to antimicrobial peptides (Bengoechea and Skurnik 2000); *kpnEF* (SMR), linked to resistance to cephalosporins and tetracyclines (Srinivasan and Rajamohan 2013); *mdtABC* (RND) (Nishino et al. 2007), involved in resistance to aminocoumarins; and *emrB-tolC* (MFS), associated with fluoroquinolone resistance (Gu et al. 2021).

With respect to enzymatic antibiotic inactivation, 13 genes were identified, but their distribution was restricted to specific species. Most of these genes correspond to β-lactamases active against cephalosporins, found in *P. ananatis*, *P. stewartii*, *P. allii*, *P. agglomerans*, *P. dispersa*, and *P. eucrina*. Additional clinically relevant enzymes included *fosA*, conferring resistance to fosfomycin, present in *P. vagans* and *P. agglomerans*, and *PSZ-1*, a β-lactamase originally reported in *P. endophytica* and here confirmed to be present in all genomes of this species, as well as its sister species, *P. trifolii*.

### Genetic determinants of ***Pantoea*** lifestyles

The genus *Pantoea* is well known for its ecological versatility, comprising strains with diverse lifestyles, including environmental, plant-associated, and even animal and human-associated forms. We classified the analyzed strains into three main groups: environmental, phytopathogenic, and clinical. Within the environmental group, endophytic species are particularly noteworthy, as they colonize plant tissues without causing visible symptoms (Hallmann et al. 1997). However, the boundary between endophytic and phytopathogenic bacteria is often blurred, since both share similar genetic repertoires, especially virulence factors, involved in plant colonization and infection (Lòpez-Fernàndez et al. 2015). This genetic overlap complicates the identification of truly beneficial strains and represents a key challenge when screening microorganisms for biotechnological applications.

**Fig. 6:**
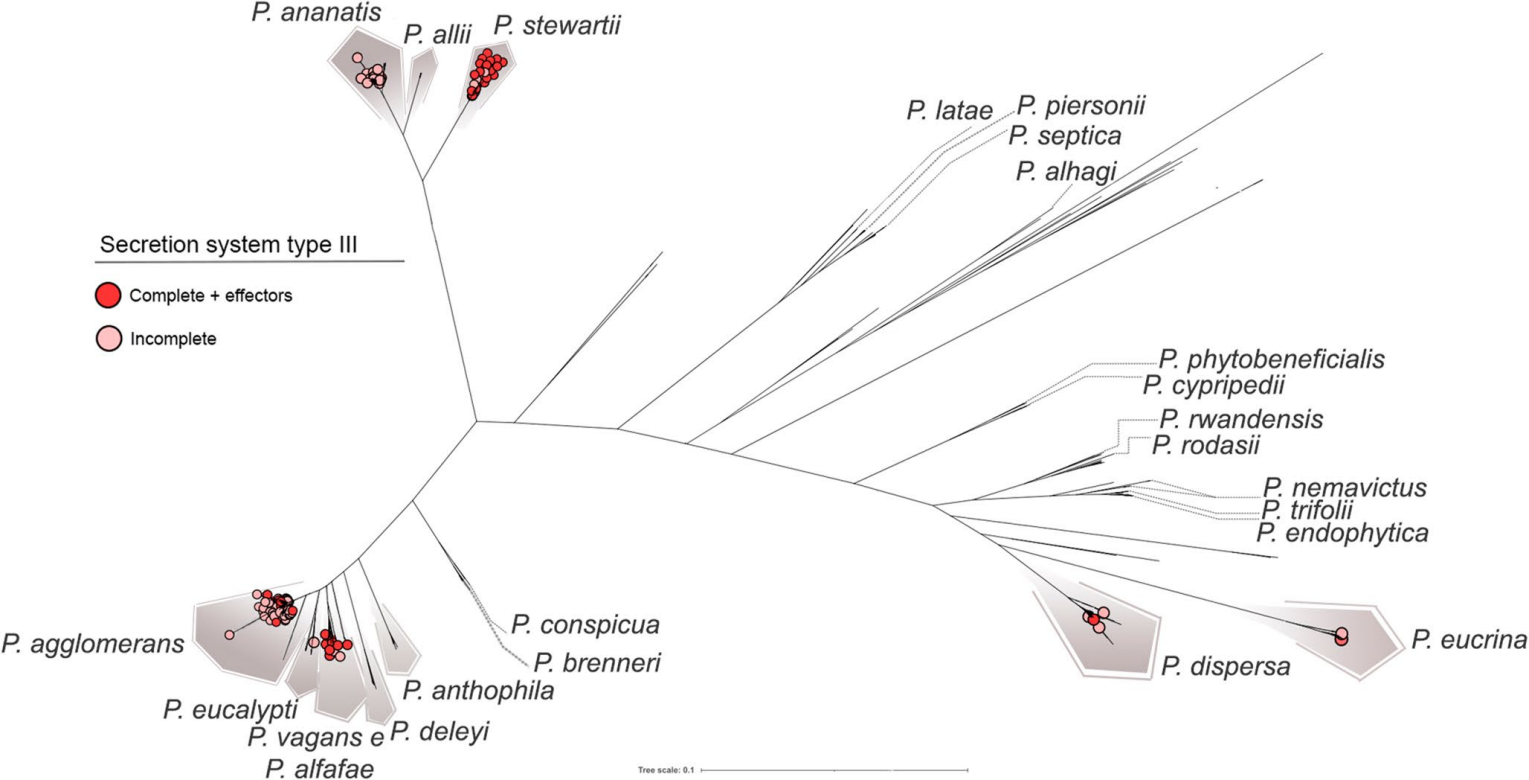
Distribution of the type III secretion system (T3SS) of the *hrp-hrc* family across *Pantoea* species. Dark red circles denote strains carrying the complete *hrp-hrc* cluster together with its effectors, while light red circles represent strains lacking the full cluster and associated effectors. The complete system is mainly found in *P. stewartii*, *P. agglomerans*, *P. vagans*, and *P. alfalfae*, which harbor all genes encoding the T3SS apparatus as well as the effectors linked to host specificity and pathogenicity—for example, *wtsE* in *P. stewartii* (water‑soaking symptoms), and *hsvB* and *hsvG* (host specificity and gall formation). The restricted distribution of the T3SS among phytopathogenic species suggests its potential as a genetic marker to distinguish pathogenic from beneficial *Pantoea* strains

**Fig. 7:**
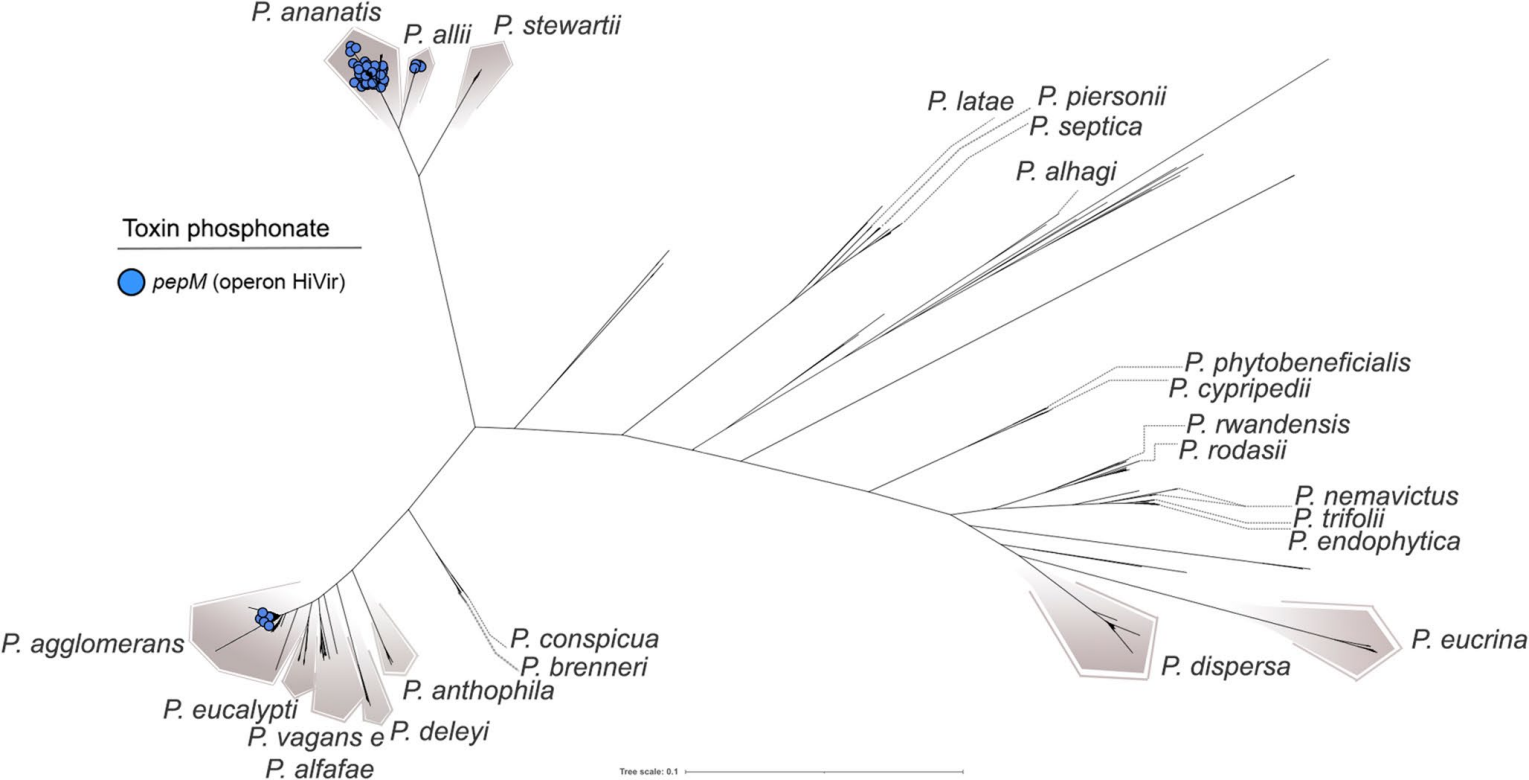
Distribution of the *pepM* gene across species of the genus *Pantoea*. The *pepM* gene is part of the HiVir operon, which encodes enzymes for the biosynthesis of pantaphos, a phosphonate phytotoxin implicated in plant disease. Species harboring *pepM* are highlighted, illustrating its restricted distribution and potential association with virulence and host specificity

To further explore these distinctions, we performed a pan-GWAS analysis to identify accessory genes associated with the different lifestyles and pinpoint genetic determinants that could serve as discriminators among the categories. Genes significantly associated with the phytopathogenic lifestyle (see Methods for details) included components of the T3SS, specific T3SS effectors, and the central gene of the phosphonate biosynthetic cluster (HiVir). The restricted distribution of these determinants among phytopathogenic strains is shown in Figs. 6 and 7.

A key determinant of pathogenicity in Gram-negative phytopathogens is the T3SS, a membrane-embedded nanomachine composed of ~ 20 structural proteins. The T3SS is encoded by the *hrp/hrc* gene cluster, whose distribution appears to be restricted to a limited number of species (Fig. 6). This system mediates the translocation of effector proteins into plant host cells through a needle-like pilus structure, known as the Hrp pilus. Once inside the host cells, these effectors can either trigger plant immune responses or suppress host defenses to promote disease development (He et al. 2025).

Among the analyzed species, *P. stewartii* harbored the complete *hrp/hrc* cluster in most genomes. The cluster was also detected in five *P. vagans* strains (plasmid-borne in one of them) and in the pathovars *P. agglomerans* pv. *gypsophila* and pv. *betae*. *P. stewartii* subsp. *stewartii* is a well-known pathogen causing Stewart’s bacterial wilt and leaf blight in maize (*Zea mays* L.), with the *hrp/hrc* cluster being essential for its pathogenicity (Mergaert et al. 1993). In *P. agglomerans*, these pathovars are well-known pathogens of *Gypsophila* and sugar beet, respectively.

Consistent with observations on phytohormone production, the *hrp/hrc* operon is also associated with gall formation, acting synergistically with auxin biosynthesis genes of the IAM pathway (*iaaM* and *iaaH*). In several instances, these determinants were plasmid-encoded, suggesting the horizontal acquisition of pathogenicity islands that enabled typically endophytic strains to adopt a pathogenic lifestyle.

Among the effectors, *wtsE* stands out as essential for lesion development in maize by *P. stewartii*, where it induces water-soaking and facilitates bacterial proliferation (Jin et al. 2016). This effector was also detected in *P. agglomerans* pv. *gypsophila* and pv. *betae*. Along with this, the effectors *hsvG* and *hsvB* were exclusively detected in these pathovars, consistent with their specialized interactions with *Gypsophila* and sugar beet.

Recent reports have also described plant lesions caused by *P. vagans* (Rodríguez Velázquez et al. 2024; Anwer et al. 2025), a species characterized by diverse lifestyles. Some of its strains, such as *P. vagans* C9-1, are recognized for their biotechnological applications and use as biocontrol agents (Klein et al. 2017), further highlighting the dual ecological roles of this species.

However, not all *Pantoea* phytopathogens rely on the T3SS. A notable exception is *P. ananatis*, widely studied for its role in onion center rot (Gitaitis et al. 2002; Stice et al. 2018; Shin et al. 2023). Unlike other pathogenic species in the genus, *P. ananatis* lacks the T3SS and instead depends on the HiVir biosynthetic gene cluster, which encodes the phosphonate phytotoxin pantaphos (Agarwal et al. 2021). Within this cluster, *pepM*, encoding a phosphoenolpyruvate mutase, is essential for pathogenicity in onions (Yang et al. 2025). This phytotoxin is predominantly found in *P. ananatis* (Fig. 7), where it is located in genomic islands, suggesting acquisition via HGT. Additionally, *pepM* was identified in four *P. allii* strains (three from onion bulbs showing center rot symptoms), one *P. vagans* strain isolated from asymptomatic *Alliaria petiolata* (Shin and Kvitko 2024), and seven *P. agglomerans* strains also isolated from diseased onions. In all cases, the genes were located within genomic islands, indicating possible plasmid-mediated transmission of the HiVir cluster across species within the genus.

**Fig. 8:**
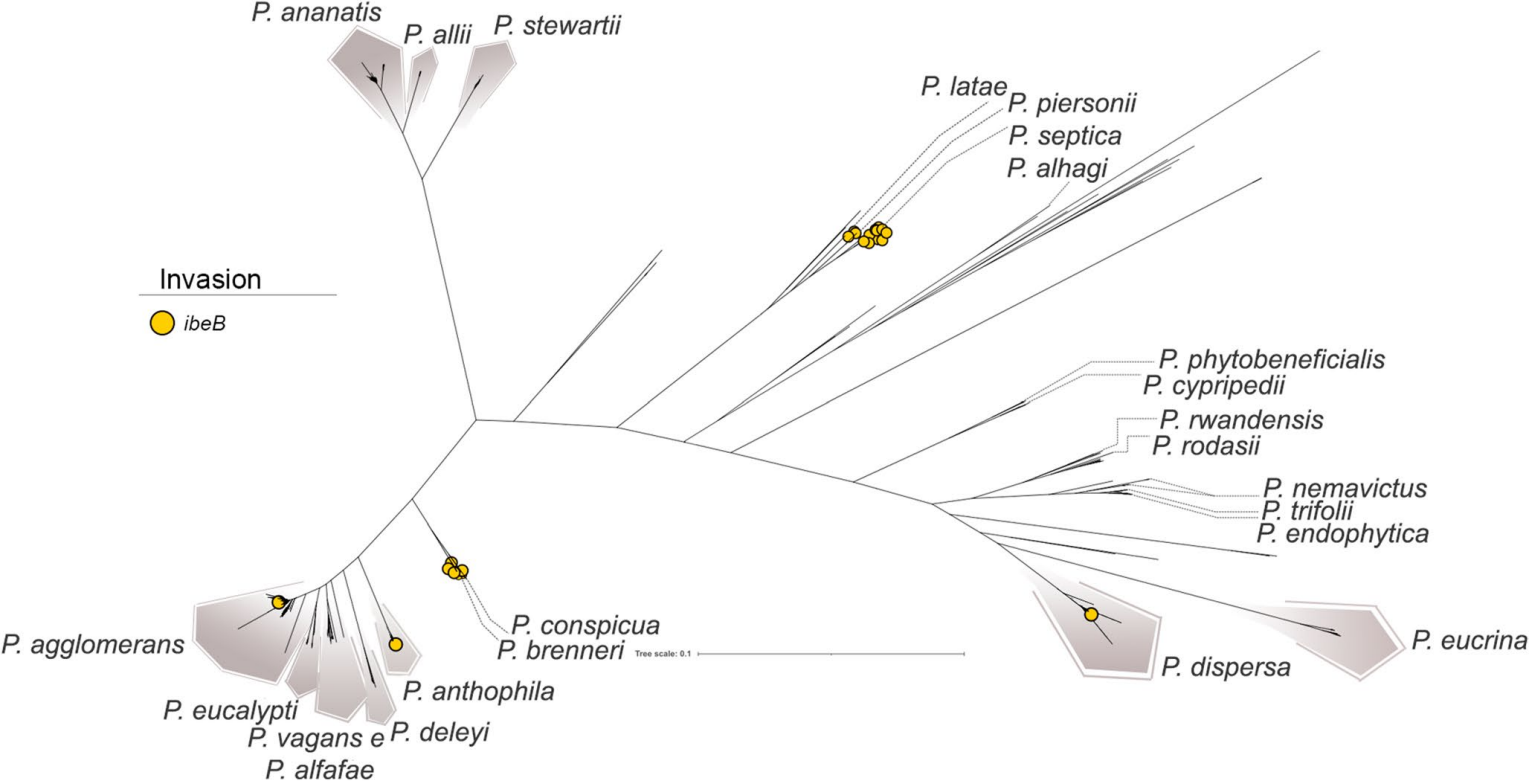
Distribution of the *ibeB* gene across *Pantoea* species, highlighting its prevalence in strains associated with clinical or pathogenic lifestyles. The presence of *ibeB* suggests a potential role in invasiveness and may serve as a marker for distinguishing pathogenic from environmental isolates

The pan-GWAS analysis identified a single gene with strong biological plausibility that was significantly associated with clinical strains: *ibeB* (also known as *cusC*), encoding an invasin-related protein. In *E. coli*, ibeB has been described as a virulence factor involved in human neonatal meningitis (Germon et al. 2005), while in *Cronobacter sakazakii*—an opportunistic pathogen responsible for severe neonatal infections such as necrotizing enterocolitis, meningitis, and sepsis—it has been similarly linked to pathogenesis (Kucerova et al. 2010). The *ibeB* gene encodes a component of a silver and copper cation efflux system, and its activity has been associated with bacterial invasiveness. In *E. coli*, for instance, ibeB facilitates the entry of bacteria into brain microvascular endothelial cells, thereby promoting penetration of the blood-brain barrier (Franke et al. 2003). This gene was most frequently detected in *P. septica*, particularly in strains isolated from neonatal feces. This species has been associated with neonatal bloodstream infections, mirroring the pathogenic profiles of *E. coli* and *C. sakazakii*, and suggesting a recurring pattern of neonatal infection. The gene was also identified in other clinically derived *Pantoea* species, including *P. piersonii*, isolated from a bacteremia case (Howard et al. 2023) and kidney stones (Rekha et al. 2020), *P. dispersa*, associated with bloodstream infections. Notably, *ibeB* was also detected in strains obtained from asymptomatic patients, underscoring its presence beyond overt clinical disease (Fig.  8).

## Discussion

In this study, we aimed to identify the main *Pantoea* species with potential for plant growth promotion and low biotechnological risk, distinguishing them from phytopathogenic and clinical strains. The ANI analysis proved sufficient to delimit species, forming well-defined groups supported by the 95% threshold. This result is particularly relevant given the broad ecological plasticity of *Pantoea*, a genus that inhabits environments ranging from plant tissues to clinical settings. Bacterial genera with such versatility often form “species complexes”—sets of distinct but genetically close lineages that coexist sympatrically and exchange genes intensively (Georgiades and Raoult 2011), as exemplified by the *Burkholderia cepacia* complex (Jia and Lu 2024).

The delimited species display striking ecological heterogeneity, with some species predominantly associated with plant lesions, while others occurring in clinical or neutral environments. *P. agglomerans*, for instance, comprises mostly endophytic and beneficial strains, whereas *P. stewartii* exhibits a clearly phytopathogenic profile. This differentiation reflects functional specialization processes mediated by HGT, which drive adaptation to specific niches.

Genome mining revealed that classical plant growth–promoting mechanisms—such as phosphate solubilization, siderophore synthesis, and phytohormone production—are broadly distributed among *Pantoea* species, corroborating its history as a plant endophyte. However, more specialized functions, such as nitrogen fixation and ACC deaminase production, were restricted to a phylogenetically cohesive group comprising *P. cypripedii*, *P. phytobeneficialis*, and two undescribed species. This restriction suggests a punctual HGT event, in which a nitrogen-fixation operon was incorporated into the chromosome. Among all species analyzed, this group exhibits the greatest biotechnological potential by concentrating key PGPB traits. Nevertheless, distinguishing endophytic from pathogenic or clinical lineages remains crucial to enable the safe exploitation of other species with similar potential but ecological heterogeneity, including pathogenic and clinical strains. Many pathogenic lineages also displayed enrichment in plant growth–promoting genes (Figs. 2 and 3), revealing a functional ambiguity—traits typically linked to mutualism may, under certain conditions, contribute to virulence.

A paradigmatic example is phytohormone production. Although auxin is classically associated with plant growth promotion, its synthesis through the IAM pathway, mediated by *iaaM* and *iaaH*, can induce tumor formation, as observed in *P. agglomerans* pv. *betae* and pv. *gypsophilae* (Barash and Manulis-Sasson 2009). The same duality applies to siderophores: while often associated with beneficial interactions, they are also vital for competition and survival in hostile environments. The predominance of the aerobactin cluster in clinical and phytopathogenic species exemplifies this duality. In human and animal pathogens such as *E. coli* and hypervirulent *Klebsiella pneumoniae*, aerobactin functions as an essential virulence factor (Pakbin et al. 2021; Lim et al. 2025), whereas in plant-associated bacteria, it may mediate competitive colonization without causing disease (Choi et al. 2022).

In our pan-GWAS analysis, aerobactin and antimicrobial resistance genes showed no significant association with pathogenic lifestyles, suggesting that these determinants are more related to basal adaptation and competition than to clinical specialization. This pattern contrasts with that observed in *Alcaligenes*, where clinical strains exhibited strong enrichment of resistance genes within genomic islands, reflecting HGT-driven specialization to hospital environments (Pedrosa-silva and Venancio 2023).

Thus, resistome evaluation in *Pantoea* played a dual role: beyond elucidating its ecological evolution, it allowed estimation of biotechnological risks. Although the genus harbors efflux pumps and inactivation enzymes—such as β-lactamases and *fosA*—these mechanisms are not uncommon in soil and rhizosphere bacteria (Delgado-Baquerizo et al. 2022) and their presence does not necessarily imply elevated clinical risk. Phenotypic susceptibility assays confirm the broad sensitivity of most strains, with resistance limited to a few antibiotics such as ampicillin, fosfomycin, and piperacillin/tazobactam (Casale et al. 2023). These findings support the safety of *Pantoea* for controlled agricultural applications.

Conversely, the analysis of virulence factors revealed determinants that may serve as biosafety markers. The complete T3SS along with *hrc/hrp* was identified exclusively in major phytopathogenic strains—*P. stewartii* and the pathovars of *P. agglomerans*—being essential for the delivery of specific effectors such as *wtsE*, *hsvB*, and *hsvG*. In other strains, part of the system was detected, suggesting loss or adaptive modification (Mengistu 2020). This pattern indicates distinct evolutionary trajectories: whereas *P. stewartii* represents a highly specialized pathogen, *P. agglomerans* pathovars appear to be in transition between endophytic and pathogenic lifestyles, possibly due to recent acquisition of virulence genes.

Pan-GWAS results showed a significant association between T3SS genes and the phytopathogenic lifestyle. Nonetheless, the mere presence of this system does not define a pathogenic phenotype. In mutualistic genera such as *Rhizobium* and *Bradyrhizobium*, the T3SS mediates beneficial interactions through Nop effectors, while in nonpathogenic *Pseudomonas* species like *P. marginalis* SBW25, it can even help promote plant growth (Zboralski et al. 2022). Hence, the functional impact of the T3SS must be interpreted within each species ecological and molecular context.

Beyond the T3SS, other genetic determinants appear equally relevant in shaping pathogenic potential within the genus. *P. ananatis* exemplifies this distinction: unlike other phytopathogenic species, it lacks a T3SS, and its pathogenicity is linked instead to the HiVir gene cluster, responsible for the biosynthesis of the phosphonate phytotoxin pantaphos. The *pepM* gene, the core element of this cluster, has been demonstrated to be essential for onion lesion formation (Yang et al. 2025), and was detected here primarily in *P. ananatis*, as well as in some *P. allii* and *P. agglomerans* strains isolated from symptomatic plant tissues. The localization of the HiVir cluster within genomic islands supports its acquisition through HGT, suggesting that pantaphos-mediated pathogenicity arose from recent gene mobilization events within the genus. This pattern underscores the remarkable plasticity of *Pantoea*, wherein different species have evolved distinct molecular strategies to interact with their hosts—some retaining the classical T3SS, while others have replaced it with alternative secretion systems and toxins.

In the clinical context, however, pan-GWAS identified *ibeB* as the main gene associated with human-derived isolates. Originally described in *E. coli* and *C. sakazakii* as a virulence factor involved in endothelial invasion and blood–brain barrier traversal (Franke et al. 2003; Kucerova et al. 2010), *ibeB* was also detected in our study within plasmids and genomic islands of *P. septica*, a species frequently linked to neonatal infections. This genomic distribution strongly suggests HGT events enabling originally commensal bacteria to acquire human-host infection potential.

Although *ibeB* alone is likely insufficient to cause infection—as indicated by its presence in asymptomatic hosts—its high frequency in *P. septica*, particularly in strains associated with neonatal sepsis, points to a potentially important role in this species’ virulence. The pathogenic mechanisms of *P. septica* remain poorly characterized, but our results highlight *ibeB* as a candidate determinant of human disease, especially in neonates. Moreover, *ibeB* was also detected in other clinically derived species, including *P. piersonii*—isolated from bacteremia and kidney stone cases (Rekha et al. 2020; Howard et al. 2023)—as well as in *P. dispersa* and *P. agglomerans*, both associated with bloodstream infections. However, the relationship between these species and human disease remains uncertain (Crosby et al. 2023). Our analyses found no unique genetic markers supporting a direct link with pathogenicity in these groups, suggesting that the transition from commensalism to opportunism depends on specific host interactions and secondary virulence factors.

Altogether, these findings reinforce that, within *Pantoea*, the acquisition of mobile genetic elements such as *ibeB* contributes to ecological versatility, facilitating the colonization of new hosts without necessarily resulting in a constitutive pathogenic phenotype.

Collectively, our results highlight the diverse evolutionary trajectories shaping *Pantoea* pathogenicity. Whereas phytopathogenic lineages appear to have evolved through specialization and acquisition of plant-specific secretion systems or toxins, clinical strains seem to rely on genetic determinants that promote survival and invasion in animal hosts. The genus therefore exemplifies an ecological continuum in which distinct gene sets (e.g., T3SS, HiVir, and *ibeB*) have emerged as independent adaptive solutions for colonizing new hosts and occupying diverse ecological niches.

Understanding the dynamics of these determinants is crucial not only for elucidating the evolution of *Pantoea* pathogenicity but also for ensuring the safe and effective use of plant growth–promoting strains. Versatile genera such as *Pantoea* pose a particular challenge, as they often couple PGPB-associated genes with potentially hazardous virulence and resistance factors—a concern emphasized by studies highlighting hidden pathogenic traits in bacterial inoculants (Tariq et al. 2022). Consequently, integrating comparative genomics with biosafety assessments is essential to differentiate genuinely beneficial strains from those carrying potential sanitary or ecological risks, thereby enabling the biotechnological application of *Pantoea* to advance in a sustainable, responsible, and safe manner.

## Conclusions

In this comparative genomics study, we evaluated the biotechnological potential of the genus *Pantoea* and identified species that appear both promising and safe for practical applications, based on the distinctions among ecological lifestyles. *P. cypripedii* and *P. phytobeneficialis* stand out, as they lack genetic determinants linked to phytopathogenicity or human virulence, while harboring a myriad of key plant growth-promoting traits, including phosphate solubilization, phytohormone production, and iron acquisition. These features underscore their potential as safe bioinoculants. Other species displayed relevant PGPB mechanisms but lacked clear ecological specialization, having been recovered from diverse environments, including insects, urban habitats, and aquatic sources. Some of these species have also been reported in pathogenic or clinical contexts, underscoring the need for further studies to properly balance their beneficial potential versus possible risks.

Within phytopathogenic groups, *P. stewartii* emerges as a genuine pathogen. In contrast, lineages of *P. agglomerans* (including pathovars *betae* and *gypsophilae* and those associated with onion center rot), as well as *P. ananatis* and *P. allii*, represent lineages that acquired phytopathogenic capacity over time. In the clinical context, *P. septica* was particularly notable due to the presence of the *ibeB* gene, a candidate determinant of neonatal sepsis that sheds light on its potential virulence mechanisms.

Taken together, our findings highlight the remarkable ecological versatility of the genus *Pantoea*, clarify the functional roles of its major species, and establish a framework for the safe and informed use of selected strains in agricultural biotechnology. At the same time, they emphasize the need for more detailed investigations into clinical and pathogenic lineages to better delineate both the risks and opportunities associated with their practical applications.

## Supplementary Information

Below is the link to the electronic supplementary material.

Supplementary Material 1 (XLSX 52.9 KB)

## Data Availability

No datasets were generated or analysed during the current study.
